# Radar and Visual Odometry Integrated System Aided Navigation for UAVS in GNSS Denied Environment

**DOI:** 10.3390/s18092776

**Published:** 2018-08-23

**Authors:** Mostafa Mostafa, Shady Zahran, Adel Moussa, Naser El-Sheimy, Abu Sesay

**Affiliations:** 1 Department of Geomatics Engineering, University of Calgary, Calgary, AB T2N 1N4, Canada; amelsaye@ucalgary.ca (A.M.); elsheimy@ucalgary.ca (N.E.-S.); 2 Department of Electrical Engineering, Port-Said University, Port Said 42523, Egypt; 3 Department of Electrical and Computer Engineering, University of Calgary, Calgary, AB T2N 1N4, Canada; sesay@ucalgary.ca

**Keywords:** multi-sensor fusion, regression trees, GNSS, EKF, UAVs, INS, RO, VO

## Abstract

Drones are becoming increasingly significant for vast applications, such as firefighting, and rescue. While flying in challenging environments, reliable Global Navigation Satellite System (GNSS) measurements cannot be guaranteed all the time, and the Inertial Navigation System (INS) navigation solution will deteriorate dramatically. Although different aiding sensors, such as cameras, are proposed to reduce the effect of these drift errors, the positioning accuracy by using these techniques is still affected by some challenges, such as the lack of the observed features, inconsistent matches, illumination, and environmental conditions. This paper presents an integrated navigation system for Unmanned Aerial Vehicles (UAVs) in GNSS denied environments based on a Radar Odometry (RO) and an enhanced Visual Odometry (VO) to handle such challenges since the radar is immune against these issues. The estimated forward velocities of a vehicle from both the RO and the enhanced VO are fused with the Inertial Measurement Unit (IMU), barometer, and magnetometer measurements via an Extended Kalman Filter (EKF) to enhance the navigation accuracy during GNSS signal outages. The RO and VO are integrated into one integrated system to help overcome their limitations, since the RO measurements are affected while flying over non-flat terrain. Therefore, the integration of the VO is important in such scenarios. The experimental results demonstrate the proposed system’s ability to significantly enhance the 3D positioning accuracy during the GNSS signal outage.

## 1. Introduction

Over the past 10 years, Unmanned Aerial Vehicles (UAVs have become an essential tool for wide civil and military applications, such as first aid delivery, firefighting, rescue, downtown traffic monitoring, disaster management, border mentoring, and reconnaissance. The deployment of these UAVs in such aspects can help minimize human exposure to hazards and risks in dangerous circumstances. Most of the UAVs are commonly dependent on their onboard Global Navigation Satellite System (GNSS)/Inertial Navigation System (INS) integrated navigation systems to estimate where they are flying while performing their missions. The main challenge for this system configuration while flying in such cluttered environments is that the satellite signals might be disturbed in various scenarios, such as signals blockage, attenuation, multipath effects, jamming, spoofing in hostile areas, and flying in urban areas or natural canyons. In such harsh scenarios, the navigation solution is obtained only by the INS prior to the GNSS signal retrieval. Hence, the navigation solution will deteriorate rapidly during this outage period due to the accumulated INS drift errors. Therefore, the incorporation of other aiding sensors is essential for mitigating the associated drift errors in INS measurements. A variety of navigation systems in GNSS denied environments have been developed by researchers to attempt an accurate navigation solution.

Visual sensors have been introduced as a navigation system for UAVs in GNSS denied environments in [1,2,3]. Due to the advancement in technology, onboard cameras have been evolved to have a small size, lightweight and, low power consumption. Furthermore, they provide valuable measurements in term of color and texture, which are used to improve the navigation solution accuracy during the GNSS outage periods. Although cameras have such great benefits, their measurements are affected by the lighting conditions, brightness, and featureless areas. Visual Odometrey, VO is a process of estimating the motion of a camera in real time using sequential images. This process is mainly classified into the monocular VO and the stereo VO. Due to the low cost and small size, the monocular VO has been widely introduced in research work [4,5,6,7,8,9,10]. The main drawback of the monocular VO is the scale ambiguity, which can make the performance of the navigation solution degrade rapidly.

To avoid this problem, a stereo VO with an overlapping field of view has been proposed in [11,12,13]. The estimated solution accuracy from such a configuration is mainly affected by slight variation on its lever arm and boresight calibration parameters during the flight. Other proposed methods integrate the monocular VO with the Inertial Measurement Unit (IMU) to recover the scale and to enhance the navigation solution [14,15].

Radar received a number of investigations during the first and second world wars due to its capability of providing warning about hostile aircrafts. After these wars, a lot of research work has been accomplished to develop more sophisticated radars. The possibility of utilizing radar for navigation purposes has been explored over the last decades. The accuracy of the radar aided navigation solution is affected mainly by the inaccuracies of radar’s Doppler measurements during this period [16]. Furthermore, the deployment of radar as part of a navigation system for UAVs is restricted due to its large size, heavy weight, high power consumption, and expensive cost. Nowadays, progress in microwave integrated circuit manufacturing technology has introduced a new miniaturized generation of Frequency Modulated Continuous Wave (FMCW) radars. In addition to miniaturization, they have low power consumption and improved measurements accuracy, which makes them more promising to be utilized in various mobile mapping and navigation applications for UAVs. Moreover, these radars can operate in diverse weather and lighting conditions.

A simulation for a navigation system in GNSS denied environments for UAVs based on ultra-wideband orthogonal frequency division multiplexed (UWB-OFDM) radar measurements is proposed by Kauffman in [17]. The tracking process is performed based on the Global Nearest Neighbor (GNN) tracker algorithms [18]. The Navigation solution is then obtained by fusing the radar range measurements with the INS via an Extended Kalman Filter (EKF).

Another Radar Odometrey (RO) aided inertial navigation system is presented by Quist in [19] for an unmanned aircraft (Cessna aircraft) in a GNSS denied environment. A Synthetic-Aperture Radar (SAR) is installed on a fixed-wing aircraft with a side-looking orientation. A pre-filtered image and Hough transform [20] based approaches are applied for the ground target detection purpose. These detected targets are then exploited to estimate the long-track and cross-track velocities over time. The height above the ground is obtained from the first range bin measurement in a range of compressed images. The estimated velocities and height are then fused with the INS measurements in an EKF. The proposed scheme is assessed through simulated data. In addition, the flight scenario is constrained by different assumptions, such as straight flights, constant velocities, leveled flights, and flying over flat terrain. Quist [21] presented a generic navigation system for UAVs in GNSS denied environments by flying in a more generic trajectory rather than in the constrained one in [19]. The estimated velocity and height from the RO, heading angle from the magnetometer, and estimated turn rate are fused with INS measurements. The performance of the proposed system is evaluated in simulated non-straight flight trajectories with different banking angles. This RO is assessed in a real flight for one minute, where an SAR radar is mounted on a Cessna aircraft. An expensive navigation-grade IMU is integrated with the SAR radar measurements to provide a more accurate navigation solution. To mimic the consumer-grade IMU performance, biases and white noise are added for the navigation-grade IMU measurements.

One RO aided navigation in GNSS-denied environments based on range progression is presented by Quist in his research work [22]. A Recursive Random Sample Consensus (RANSAC) algorithm is developed for target detection, tracking, and range rate estimation purpose. The heading from the magnetometer, pseudo turn rate, range to the scatterers’ measurements, relative range, and altitude above the ground are utilized as measurement updates for the navigation-grade IMU in an EKF. The proposed system is tested in a real flight data set with an SAR mounted on a Cessna aircraft.

Another RO based approach has been proposed by Scannapieco for small and micro UAVs in cluttered environments in [23]. A radar is attached to the UAV to provide range and azimuth measurements for ground targets. The Constant False Alarm Rate (CFAR) filter [24] is employed for the target detection task while the multiple-target tracker (MTT) algorithm is utilized for the tracking process. A Singular Value Decomposition (SVD) algorithm [25] is applied to the tracked objects to estimate the relative translation and rotation of the UAV. Tightly and loosely coupled techniques have been used to integrate the RO with the INS measurements in an EKF. The range and bearing are utilized as a measurement update for the tightly coupled approach, while the estimated pose variation and heading angle from the RO are utilized to update the loosely coupled EKF. Two flights are performed in different places with multiple radar configurations to assess the proposed system performance. Although this system provides a navigation solution in GNSS denied environments, it is unable to distinguish between rotation and translation, especially when the vehicle rotates without any translation or with a small movement.

Light Detection and Ranging (LiDARs) sensors are employed in some navigation systems for UAVs in GNSS denied environments [26,27]. In such navigation systems, LiDARs sensors are utilized to generate a digital elevation model (DEM) and match it to previously stored ones. The main limitation of these LiDAR sensors for outdoor environments is that their measurements are affected by the environmental conditions, such as rain and fog.

This paper presents an accurate integrated system (RO/VO/magnetometer/barometer) aided navigation system for small UAVs in GNSS denied environments. The main contribution of the proposed system is that it significantly improves the 3D positioning accuracy during the GNSS signal outages by accurately estimating the forward vehicle velocity from both the RO and enhanced VO. The incorporation of the RO and VO into an integrated system helps overcome their limitations. The RO is immune against the environmental conditions, such as rain, fog, and dust. Unlike the camera, it is not affected by the change of the illumination or featureless area. On the other hand, the RO measurements are affected while an aircraft is flying over multiple objects with different altitudes, ranges, and angles. The utilized radar does not provide azimuth and elevation measurements for the observed objects with different angles values inside the radar beam, and the radar tilt angle (60°) is the only utilized angle to estimate the vehicle forward velocity. Therefore, the accuracy of the estimated forward velocity from the RO is downgraded while the aircraft is flying over non-flat terrain. Therefore, the incorporation of the camera in such challenging scenarios can help to enhance the navigation solution.

Furthermore, the scale ambiguity of proposed monocular VO is resolved based on the radar height measurements. In addition, a small SOLO quadcopter is equipped with the camera and micro radar, which is typically utilized in many missions, such as search, rescue and disaster management. The computational time for the target detection process is 1.3 ms, which makes it convenient for real-time applications. Moreover, the proposed RO exploits ground scatterers from grass, trees or any other objects in the surrounding environment for the velocity estimation, rather than relying on artificial reflectors. Additionally, the proposed VO is enhanced with a regression tree-based approach in an attempt to correct the estimated forward velocity from the VO drift errors.

This paper is organized as follows: Section 2 describes the system overview of the proposed algorithm. The experimental results are presented and discussed in Section 3. Finally, the conclusions are given in Section 4.

## 2. System Overview of the Proposed Algorithm

**Figure 1:**
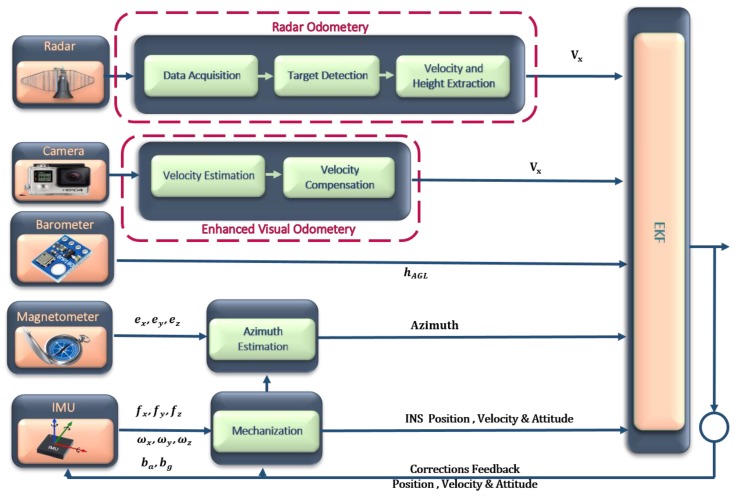
Overall block diagram for the proposed system.

An integrated navigation system for UAVs in GNSS denied environments is proposed based on the RO and enhanced VO. This system is implemented based on three major steps: the enhanced monocular VO, RO, and data fusion. The enhanced monocular VO is proposed based on the optical flow and regression trees. The optical flow is utilized to estimate the vehicle forward velocity while the regression tree algorithm is employed for the estimated velocity drift compensation purpose. On the other hand, the RO is implemented based on three steps: the data acquisition, target detection, and data extraction. The Gaussian kernel, and local maxima-based approaches are employed for the RO target detection. This system integrates the estimated forward velocities in the body frame, which are obtained from the RO, and the enhanced monocular VO with the IMU, barometer, and magnetometer measurements via an EKF. Figure 1 illustrates an overall block diagram for the proposed system.

### 2.1. Frequency Modulated Continuous Wave Radar Odometry

The proposed RO estimates the vehicle forward velocity and the height above the ground from its Range Doppler Map (RDM). This RO consists of data acquisition, target detection, velocity, and height above ground extraction.

#### 2.1.1. Data Acquisition

This RO acquires the ranges and velocity measurements for the reflected ground scatterer based on an internal radar signal processing. During the flight, the attached micro radar is continuously transmitting a frequency modulated sawtooth chirps toward the ground objects $f_{RF\;TX}$:

(1) $$f_{RF\;TX}=f_{0}+K_{f}*t,0\leq t<T$$

where $K_{f}$ is the sweep rate, T is the frequency sweep time, and $f_{0}$ is the initial transmitted frequency. This sweep rate can be obtained as follows:

(2) $$K_{f}=\frac{BW}{T}$$

where BW represents the transmitted chirp signal bandwidth. A roundtrip propagation time delay Δt and a small frequency shift between the transmitted and received signals take place due to the range propagation effect. The time propagation delay for the reflected signals from each scatterer *i* is:

(3) $$\Delta t=2\frac{r_{i}}{c}$$

where c is the speed of light, and $r_{i}$ is the range between the micro radar and each object which is located within the radar beamwidth. The originally transmitted signal is then mixed with the received signal in a low-pass filter to estimate the video signal x(t) that has a Beat frequency $f_{b}$, which can be written as:

(4) $$f_{b}=\frac{BW}{T}*2\frac{r_{i}}{c}$$

**Figure 2:**
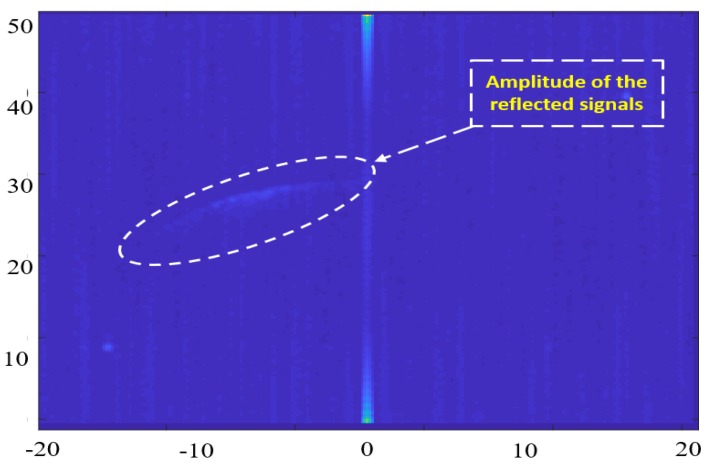
Reflected ground signals in the Range Doppler Map (RDM) image.

The phase change of this video signal is then utilized to obtain the Doppler frequency $f_{doppler}$. In each chirp, this video signal is sampled with a 264-ns sampling rate. A two-dimensional Fast Fourier Transform (FFT) is then applied on the sampled signal [28] to form the radar RDM for each of the three receiving antennae. A mean RDM is generated by averaging the RDM from all antennae. This mean RDM has 256 × 256 pixels with a 32-Bit amplitude value for each pixel, which is used to obtain the radar range and velocity measurements for different scatterers. Figure 2 illustrates an RDM image where the X-axis represents the velocity measurements and the Y-axis provides the range measurements. The 32-Bit amplitude value at each pixel represents the received echo signals strength from different ground scatterers.

This RDM is acquired from the micro radar through an Ethernet cable after the radar signal processing inside the radar is performed. This generated map is then utilized for the target detection, velocity estimation, and height extraction purposes.

#### 2.1.2. Target Detection and Data Extraction

**Figure 3:**
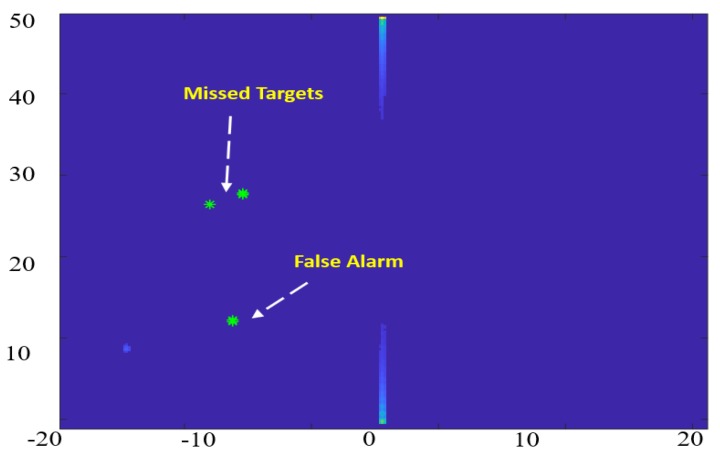
Constant False Alarm Rate (CFAR) detected targets in the RDM image.

The target detection process of radar is commonly achieved by utilizing the Constant False Alarm Rate (CFAR) algorithm. This algorithm adaptively estimates the threshold power level by locally comparing the power for the cell-under-test (CUT) against its neighborhood cells (background) [29]. The target detection is achieved when the power level of the CUT exceeds the estimated threshold level. Many ground radar stations and airborne radars are relying on the CFAR for the aircraft detection purpose. Although the CFAR is an effective detection approach for these applications, it is not effective for the proposed system since the ground scatterers have approximately the same power level on the RDM. The CFAR-detected targets for the RDM image of Figure 2 is shown in Figure 3 where CFAR could detect a part of the main reflected arc of the ground objects while it misses the rest of it. On the other hand, the CFAR can detect a false target (noise), which has a prominent power level from its background.

The first issue occurs when the CUT is picked inside a patched area of real ground scatterers. In this case, the CUT has approximately the same power level as its surrounding background and the CFAR could not detect all the targets since the CUT has the same power level as its neighborhood, which makes it not distinctive.

**Figure 4:**
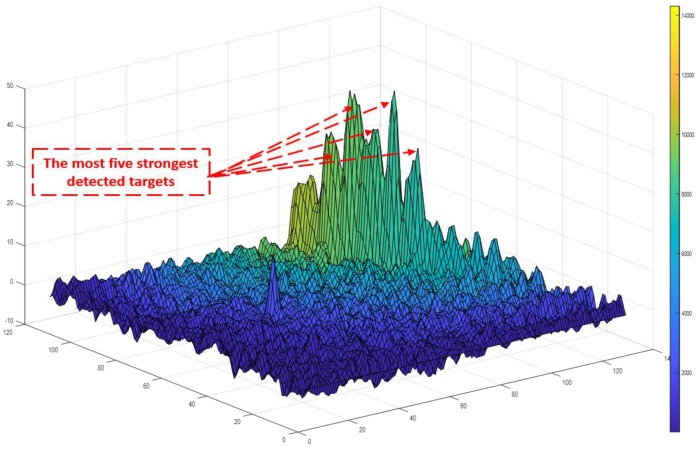
Radar detected targets.

The second issue results from random noises, which have relatively high-power levels compared to its local neighborhood cells. Unlike the CFAR, which depends on the local neighborhood cells for target detection, an efficient target detection technique is proposed based on the Gaussian kernel and local maxima to avoid problems associated with the CFAR. The Gaussian kernel is convolved with the RDM as a filtering step to depress the isolated noise responses, which may affect the RO estimated height and velocity. A local maxima-based approach is then applied as a global threshold to obtain the strongest five candidates on the whole image which are considered to be the detected targets, as shown in Figure 4. The target detection process takes approximately 1.3 ms, which is convenient for real-time applications.

The radial velocity and the range for each detected target are then acquired directly from the X- and Y-axes of this image, respectively. These radial velocities are projected by the radar tilt angle, which is 60° with respect to the flight path direction in addition to the vehicle pitch angle. These projected components are then averaged to obtain the resultant forward velocity of the vehicle in the body frame. On the other hand, the ranges are projected and compensated by the same angles with respect to the vertical direction and then averaged to obtain the height above the ground level for the vehicle.

### 2.2. Enhanced Moncular Visual Odometry

A GoPro HERO4 camera is attached to the quadcopter with a resolution of 1080 × 1920, and a video measurement rate of 30 frames per second. This camera is mounted on the UAV to have a downward facing orientation. The proposed VO consists of two main steps, which are the monocular VO based on the optical flow, and regression tree-based approach for the velocity drift error compensation.

#### 2.2.1. Monocular Visual Odometry

**Figure 5:**
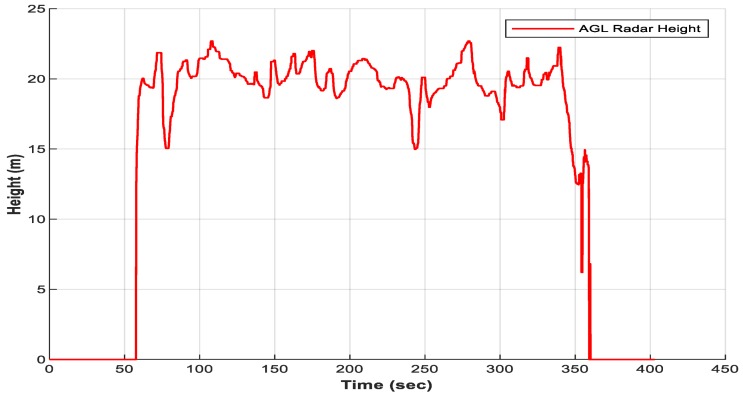
Above Ground Level (AGL) estimated height from the Radar Odometry (RO).

The proposed VO extracts the optical flow vectors in the X and Y directions by detecting the features of interest from the video frames using the Speeded Up Robust Features (SURF) detector [30]. These detected features are then matched between successive frames. The main reason for utilizing the SURF algorithm is that it has a low computational load, which makes it more convenient for real-time operation. An M-estimator Sample Consensus (MSAC) algorithm is employed for outlier (incorrect match) rejection purpose. Suppose a point P=[X,Y,Z] in the space is projected by a pinhole camera model to an image plane at point $p=\left[x_{pix},y_{pix},f_{cam}\right]$, which is described as:

(5) $$p=\frac{f_{cam}}{Z}P$$

where $f_{cam}$ is the camera’s focal length. Since the camera is mounted perpendicularly to a vehicle body, the coordinate Z is approximately equal to the distance between ground and camera’s projection origin. The estimated ground distance using radar measurement is shown in Figure 5.

By having this ground distance, the displacement in the image plane (Δu,Δv) can be converted to a real-world displacement (ΔX,ΔY) as following:

(6) $$\Delta X=-\frac{1}{f_{cam}}\Delta u.Z,\Delta Y=-\frac{1}{f_{cam}}\Delta v.Z$$

The displacement in the image plane is obtained after removing the outliers from the matched points between two successive frames. As the computed displacement (Δu,Δv) is usually in pixels, it is required to convert it into real-world units (e.g., meters), which is written as:

(7) $$\Delta X=-\frac{s}{f_{cam}}\Delta u.Z,\Delta Y=-\frac{s}{f_{cam}}\Delta v.Z$$

where s is the pixel size. The optical vectors (u,v) are obtained by multiplying these image displacements by the camera’s measurements rate. The gyro’s measurements $\omega_{x},\omega_{y}$ are then utilized to compensate the vehicle rotational motion effect from the estimated optical flow vectors in the X and Y directions. These compensated vectors are then utilized to estimate the vehicle forward velocity $V_{\mathrm{VO}}$, which is described as:

(8) $$V_{\mathrm{VO}}=-\left[\frac{s}{f_{cam}}u-f\tan\left(\omega_{y}\Delta t\right)\right]\cdot Z$$

where ∆*t* is the time between two consecutive frames.

#### 2.2.2. Velocity Compensation

During this velocity compensation phase, a regression tree-based approach is employed to enhance the accuracy of the estimated vehicle velocity from the monocular VO and compensate the employed approximations in the previous velocity estimation step. This training process is achieved during the availability of the GNSS signals. The GNSS signals are assumed to be available during the first 50 and 140 s of the first and second flights, respectively. The training process takes place during the first 40 s (from 1 to 40 s) for the first flight and the remaining 10 s (40 to 50 s) of the GNSS signals availability are utilized to obtain the weights for the predicted velocity from the regression trees. These weights are then utilized for the averaging process between the predicted velocity from the regression trees and the estimated vehicle forward velocity from the monocular VO. During the second flight, the whole collected data during the first flight and the first 125 s (from 1 to 125 s of the GNSS signal availability) of the second flight are utilized for the training purpose. The remaining 15 s (125 to 140 s of the GNSS signal availability) are utilized to obtain the weights for the predicted velocity from the regression trees.

**Figure 6:**
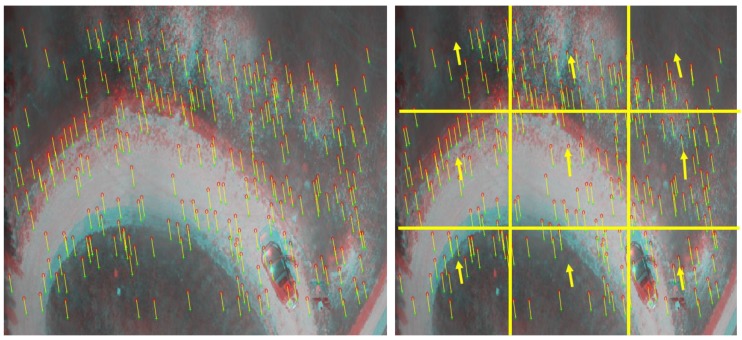
The optical flow vectors and the averaging process among 3 × 3 image cells.

**Figure 7:**
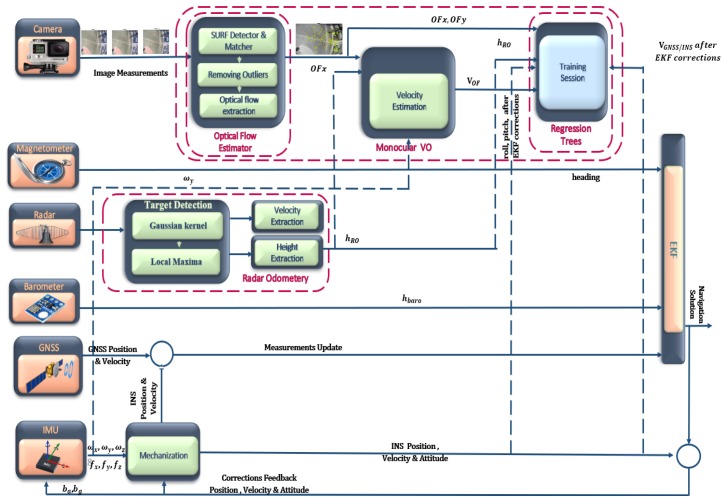
Proposed system architecture during the training session.

The video frames are divided into 3 × 3 cells, and the optical flow vectors in the X and Y directions are then averaged inside each cell to offer a fixed number of vectors to the regression trees during this stage, as shown in Figure 6. Theses averaged optical flow vectors along each cell, the estimated forward velocity from the monocular VO, roll, pitch, and RO height are then utilized as inputs for the regression trees during the training session while the ground truth forward velocity, which is obtained from the GNSS/INS/magnetometer/barometer integration, is utilized as an output during this training phase. Figure 7 illustrates the proposed system architecture during the training session.

**Figure 8:**
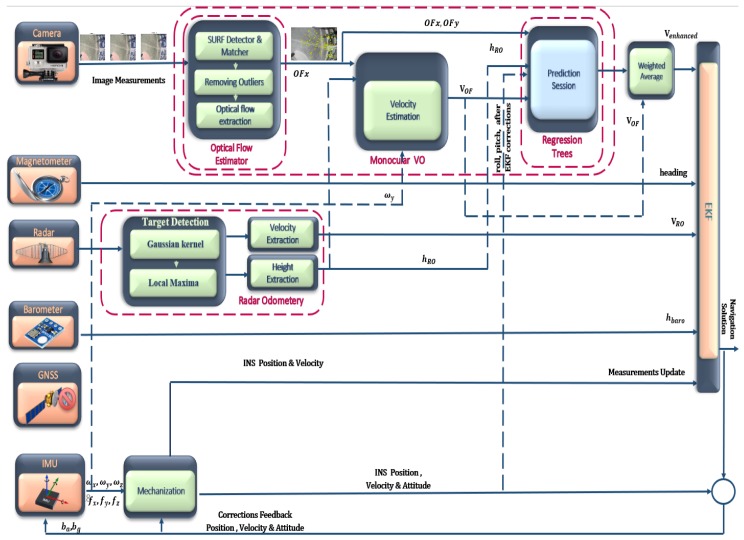
Proposed system architecture during the prediction session.

When the GNSS signals are unavailable, the trained regression trees start to estimate the vehicle forward velocity in an attempt to compensate the monocular VO drift errors caused by different reasons, such as featureless area, inconsistent matches, and the change of the camera calibration parameters due to the strong vehicle vibration effect during the flight. A weighted average between the predicted velocity from the regression trees and the estimated velocity from the monocular VO is utilized as a measurements update for the EKF. These weights are obtained by computing the Root Mean Square (RMS) errors for the predicted velocity from the regression trees and the estimated velocity from the monocular VO with respect to the reference forward velocity from 40 to 50 s for the first flight and from 125 to 140 s for the second flight. The estimated forward velocity from the RO is also utilized as a measurement update during this GNSS outage period. Figure 8 illustrates the proposed system architecture during the prediction session. The regression tree-based approach is employed since it has the ability to handle the situation of missing inputs (optical flow vectors) in some image parts due to the lack of the observed features or inconsistent matches caused by repeated patterns.

### 2.3. Data Fusion

This section describes the data fusion between INS, magnetometer, barometer, enhanced VO, RO, and GNSS measurements during its availability in an EKF. The navigation states, which are the position, velocity, and attitudes in the navigation frame (n-frame) are derived from the IMU raw measurements through a mechanization process. The EKF error states vector include 21 states as follows:

(9) $$x=\left[\begin{array}{ccc} \delta r^{n}{\;}_{1\times 3} & \delta v^{n}{}_{1\times 3} & \begin{array}{ccc} \varepsilon^{n}{}_{1\times 3} & b_{1\times 3} & \begin{array}{ccc} d_{1\times 3} & s_{a}{}_{1\times 3} & s_{g}{}_{1\times 3} \end{array} \end{array} \end{array}\right]$$

where $\delta r^{n},\delta v^{n},\varepsilon^{n}$ are the (position, velocity and attitude) error vector of INS mechanization, respectively, and b and d are the accelerometers bias and gyros drift, respectively. Finally, $s_{a}$ and $s_{g}$ are the accelerometers and gyros scale factor, respectively. The whole sensors are fused in loosely coupled fashion through two main steps inside the EKF, the prediction phase and the measurements update phase.

#### 2.3.1. Prediction Model

The system model, which describes how different INS error states evolve with time, is obtained by linearly perturbing the mechanization equations and can be represented [31,32] as follows:

(10) $${\hat{x}}_{k+1}^{-}=\Phi_{k,k+1\;}{\hat{x}}_{k}^{+}+w_{k}$$

where $\Phi_{k,k+1}$ is the state transition matrix, ${\hat{x}}_{k}^{+}$ is the error states from the previous epoch, ${\hat{x}}_{k+1}^{-}$ is the predicted error states, and $w_{k}$ is the system noise. The inertial sensor stochastic errors are modeled as a first-order Gauss–Markov process. The prediction of state-error covariance matrix $P_{k+1}^{-}$ at a certain epoch is:

(11) $$P_{k+1}^{-}=\Phi_{k,k+1}P_{k}^{+}\Phi_{k,k+1}^{T}+{\bar{G}}_{k}Q_{k}{\bar{G}}_{k}^{T}$$

where $Q_{k}$ is the covariance matrix of the system noise, $P_{k}^{+}$ is the error states covariance matrix from the previous epoch, and $G_{k}$ is the noise coefficient matrix.

#### 2.3.2. Observation Model

The differences between the GNSS positioning measurements ${\tilde{P}}_{\mathrm{GNSS}}^{\mathrm{ned}}$ and the computed positions of the GNSS antenna center ${\hat{P}}_{\mathrm{GNSS}}^{\mathrm{ned}}$, which are derived from the INS positions ${\hat{P}}_{\mathrm{IMU}}^{\mathrm{ned}}$ in the navigation frame, are utilized as measurement updates in the EKF. The measured position by the GNSS [31] can be written as:

(12) $${\tilde{P}}_{\mathrm{GNSS}\;}^{\mathrm{ned}}{}_{3\times 1}=P_{\mathrm{GNSS}}^{\mathrm{ned}}{}_{3\times 1}+D^{-1}{}_{3\times 3}{\;e}_{P}{}_{3\times 1}$$

where $e_{P}$ is the vector of GNSS position measurement errors and can be expressed as:

(13) $$e_{P}{}_{1\times 3}=\left[e_{n}\;\vdots \;e_{e}\;\vdots \;e_{d}\right]$$

where $e_{n},\;e_{e},\;e_{d}$ are the positioning errors in the North, East and Down, respectively.

(14) $$D^{-1}=\left[\begin{array}{ccc} \frac{1}{R_{M}+h} & 0 & 0\\ 0 & \frac{1}{\left(R_{N}+h\right)\cos\varphi } & 0\\ 0 & 0 & -1 \end{array}\right]$$

(15) RM=R(1−e2)(1−e2sin2φ)32

(16) RN=R(1−e2sin2φ)12

where R is the equatorial earth radius, e is the eccentricity of the earth ellipsoid, and φ is the latitude. The positioning measurements which are utilized for updating the EKF are:

(17) $$z_{\mathrm{GNSS}}=D\left({\hat{P}}_{\mathrm{GNSS}}^{\mathrm{ned}}-{\tilde{P}}_{\mathrm{GNSS}}^{\mathrm{ned}}\right)=\delta P_{\mathrm{IMU}}^{\mathrm{ned}}+\left(C_{b}^{l}l_{\mathrm{GNSS}}^{b}\times \right)\emptyset -e_{P}\;=\left[I_{3\times 3}\vdots 0_{3\times 3}\vdots C_{b}^{l}\left(l_{\mathrm{GNSS}}^{b}\times \right)\vdots 0_{3\times 12}\right]x-e_{P}$$

where the parameter $l_{\mathrm{GNSS}}^{b}\times$ is the skew-symmetric form of the lever-arm between the GNSS antenna and the IMU in the body frame, $C_{b}^{l}$ is the rotation matrix between the body and local level frame, $\delta P_{\mathrm{IMU}}^{\mathrm{ned}}$ are the positions error states, and ∅ is the attitude errors which can be expressed in a skew-symmetric form $E^{ned}$ (or cross product ∅×) form [33] as:

(18) $$E^{ned}=\emptyset \times =\left[\begin{array}{ccc} 0 & -\epsilon_{d} & \epsilon_{e}\\ \epsilon_{d} & 0 & -\epsilon_{n}\\ -\epsilon_{e} & \epsilon_{n} & 0 \end{array}\right]$$

where $\epsilon_{n},\epsilon_{e}$ and $\epsilon_{d}$ are the attitude errors in the North, East and Down directions, respectively. From Equation (17), the design matrix can be expressed as:

(19) $$H_{GNSS}{}_{3\times 21}=\left[I_{3\times 3}\;\vdots \;0_{3\times 3}\;\vdots \;C_{b}^{l}{}_{3\times 3}{\left(l_{\mathrm{GNSS}}^{b}\times \right)}_{3\times 3}\;\vdots \;0_{3\times 12}\right]$$

Finally, the innovation sequence between the measurement updates $z_{\mathrm{GNSS}}$ and the estimated measurements ${\hat{z}}_{\mathrm{GNSS}}$ is obtained as:

(20) $$\delta z_{\mathrm{GNSS}}=z_{\mathrm{GNSS}}-{\hat{z}}_{\mathrm{GNSS}}=z_{\mathrm{GNSS}}-H_{GNSS}x$$

The heading measurements $\psi_{\mathrm{mag}}$ is acquired from the magnetometer raw measurements (3D magnetic field components) as:

(21) $${\tilde{\psi }}_{\mathrm{mag}}=\tan^{-1}\left(\frac{-M_{y}\cos\Phi +M_{z}\sin\Phi }{M_{x}\cos\theta +\left(M_{y}\sin\Phi +M_{z}\cos\Phi \right)\sin\theta }\right)+\delta_{mag}+e_{\psi_{\mathrm{mag}}}$$

where $\left[\begin{array}{ccc} M_{x} & M_{y} & M_{z} \end{array}\right]$ are the unit-vector measurements of magnetic north in the body frame, Φ is the roll angle, θ is the pitch angle and $\delta_{mag}$ is the magnetic declination, which is the bearing the difference between the true north and magnetic north, and $e_{\psi_{\mathrm{mag}}}$ is the magnetometer heading measurement error.

The estimated Direction Cosine Matrix (DCM) ${\hat{C}}_{b}^{l}$, which is derived from the mechanization process during the EKF prediction stage, is utilized to compute the heading angle $\hat{\psi }$. The heading measurement, which is used for updating the EKF, is the difference between the estimated heading $\hat{\psi }$ and the measured heading ${\tilde{\psi }}_{\mathrm{mag}}$ and can be written as:

(22) $$z_{\mathrm{mag}}=\hat{\psi }-{\hat{\psi }}_{\mathrm{mag}}$$

The error equation can be obtained by perturbing Equation (22) as:

(23) $$\delta \psi =\frac{\partial \hat{\psi }}{\partial \epsilon_{n}}\epsilon_{n}+\frac{\partial \hat{\psi }}{\partial \epsilon_{e}}\epsilon_{e}+\frac{\partial \hat{\psi }}{\partial \epsilon_{d}}\epsilon_{d}$$

Hence, the design matrix can be expressed as:

(24) $$H_{mag}{}_{1\times 21}=\left[0_{1\times 6}\vdots 0_{1\times 2}\vdots 1\vdots 0_{1\times 12}\right]$$

The innovation sequence between the measurement update $z_{\mathrm{mag}}$ and the estimated measurements ${\hat{z}}_{\mathrm{mag}}$ is obtained as:

(25) $$\;\delta z_{\mathrm{mag}}=z_{\mathrm{mag}}-{\hat{z}}_{\mathrm{mag}}=z_{\mathrm{mag}}-H_{mag}x$$

The barometer is utilized to measure the height of the vehicle $h_{\mathrm{baro}}$ as:

(26) $${\tilde{h}}_{\mathrm{baro}\;}=h_{\mathrm{baro}}+e_{h_{baro}}$$

where $e_{h_{baro}}$ is the barometer height measurement error. The design matrix can be expressed as:

(27) $$H_{baro}{}_{1\times 21}=\left[0_{1\times 2}\;\vdots \;1\;\vdots \;0_{1\times 3}\;\vdots \;0_{1\times 3}\;\vdots \;0_{1\times 12}\right]$$

The innovation sequence between the measurement update $z_{baro}$ and the estimated measurements ${\hat{z}}_{\mathrm{baro}}$ is obtained as:

(28) $$\delta z_{\mathrm{baro}}=z_{\mathrm{baro}}-{\hat{z}}_{\mathrm{baro}}=z_{\mathrm{baro}}-H_{baro}\;x$$

The estimated velocity in the flight path direction, which is obtained from the RO, can be written as:

(29) $${\tilde{V}}_{\mathrm{RO}}^{s}=V_{\mathrm{RO}}^{s}+e_{V}$$

where $V_{\mathrm{RO}}^{s}$ is the velocity acquired from the RO represented in the sensor frame and $e_{V}$ is the velocity measurement noise. The difference between the predicted forward velocity from the INS in the sensor frame and the extracted forward velocity from the RO is utilized as a measurement update during the GNSS signal outage. The attitude errors and the angular rate errors must be incorporated in the derived velocity in the mechanization process. The EKF measurement updates can be expressed as:

(30) $$z_{V_{\mathrm{RO}}}={\hat{V}}_{\mathrm{RO}}^{s}-{\tilde{V}}_{\mathrm{RO}}^{s}=C_{b}^{s}C_{l}^{b}\delta V_{\mathrm{IMU}}^{\mathrm{ned}}-C_{b}^{s}C_{l}^{b}\left(V_{\mathrm{IMU}}^{\mathrm{ned}}\times \right)\emptyset -C_{b}^{s}\left(l_{RO}^{b}\times \right)\delta \omega_{\mathrm{ib}}^{b}-e_{V}$$

where ${\hat{V}}_{\mathrm{RO}}^{s}$ is the computed RO forward velocity from the INS represented in the sensor frame, $V_{\mathrm{IMU}}^{\mathrm{ned}}\times$ is the skew-symmetric form of the velocity of the vehicle at the center of the IMU represented in the navigation frame, $l_{RO}^{b}\times$ is the skew-symmetric form of the lever arm between the RO and the IMU represented in the body frame, $C_{l}^{b}$ is the rotation matrix between the navigation and body frame. $\delta V_{\mathrm{IMU}}^{\mathrm{ned}}$ is the velocity error of the vehicle at the center of the IMU and $\delta \omega_{\mathrm{ib}}^{b}$ is the angular rate measurement error. From Equation (30), the design matrix can be expressed as:

(31) $$H_{\mathrm{RO}}{}_{3\times 21}=\left[0_{3\times 3}\;\vdots \;C_{l}^{s}{}_{3\times 3}\;\vdots \;-C_{l}^{s}{}_{3\times 3}{\left(V_{\mathrm{IMU}}^{\mathrm{ned}}\times \right)}_{3\times 3}\;\vdots \;0_{3\times 3}\;\vdots \;-C_{b}^{s}{}_{3\times 3}{\left(l_{RO}^{b}\times \right)}_{3\times 3}\;\vdots \;0_{3\times 6}\right]$$

The innovation sequence between the measurement updates $z_{V_{\mathrm{RO}}}$ and the estimated measurements ${\hat{z}}_{V_{\mathrm{RO}}}$ is obtained as:

(32) $$\delta z_{V_{\mathrm{RO}}}=z_{V_{\mathrm{RO}}}-{\hat{z}}_{V_{\mathrm{RO}}}=z_{V_{\mathrm{RO}}}-H_{\mathrm{RO}}x$$

The enhanced monocular VO velocity update is derived in the same manner as the RO velocity update. After the innovation sequence $\delta z_{k}$ and design matrix $H_{k}$ computation for each sensor, the Kalman gain $K_{k}$, the updated states ${\hat{x}}_{k}^{+}$, and its updated covariance matrix $P_{k}^{+}$ can be obtained as:

(33) $$K_{k}=P_{k}^{-}H_{k}^{T}{\left(H_{k}P_{k}^{-}H_{k}^{T}+R_{k}\right)}^{-1}$$

(34) $${\hat{x}}_{k}^{+}={\hat{x}}_{k}^{-}+K_{k}\delta z_{k}$$

(35) $$P_{k}^{+}=\left(I-K_{k}H_{k}\right)P_{k}^{-}$$

## 3. Experimental Results

The results shown in the upcoming subsections are obtained from two-real flights with 3DR Solo quadcopter equipped with a micro-FMCW radar and a GoPro Hero4 camera.

### 3.1. Hardware Setup

**Figure 9:**
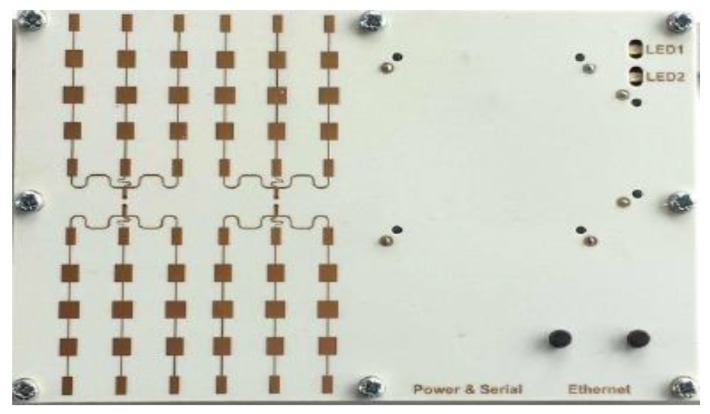
**Figure 9. **Utilized Frequency Modulated Continuous Wave (FMCW) radar (RF-beam).

A GoPro Hero4 camera with a fisheye lens is attached to the UAV to get a High Definition (HD) video measurement with 30 frames per second. Two real flights are performed in different places. During the first flight, the camera’s field of view is adjusted to be wide with a resolution of 1080 × 1920, while the field of view is adjusted to be medium with the same resolution during the second flight. A K-MD2 radar module is attached to the quadcopter belly through a wooden frame. This radar has a 24-GHz transmitted frequency with one transmitter and three receiver antennae. The radar range is measured up to 300 m with a 1-m range resolution and velocity measurements up to 40 m/s with a 0.6-m/s resolution accuracy. The UAV has a compact size as the dimensions of the radar are 120 × 72 × 15 mm, the radar weight is 165 g which makes it suitable for small UAVs, and the power consumption of this radar is 7.2 watt at 12 V. Figure 9 illustrates the utilized radar.

**Figure 10:**
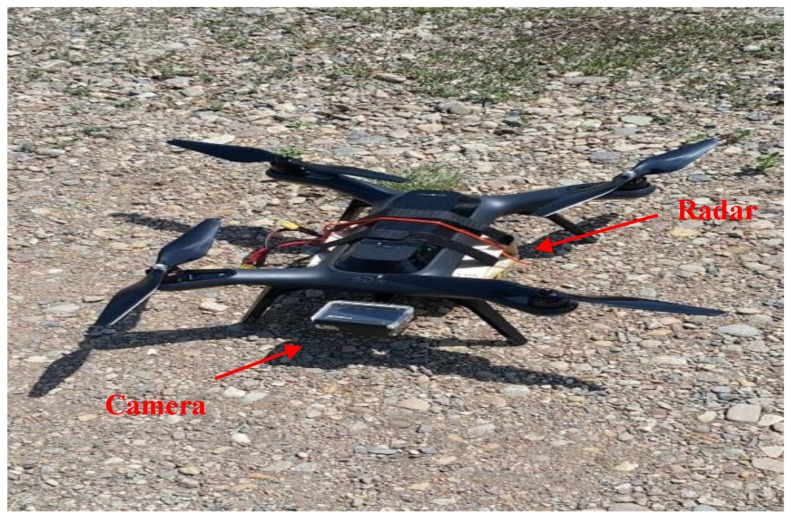
Attachment of the camera and radar to the SOLO quadcopter.

**Figure 11:**
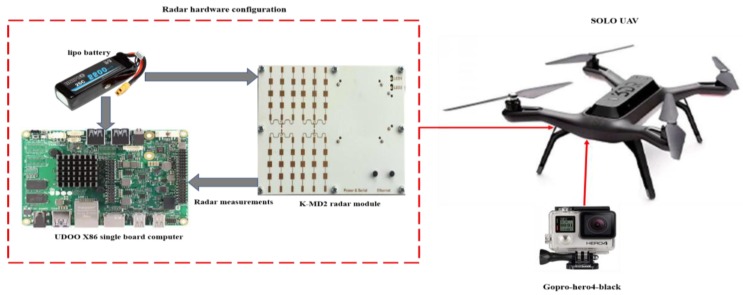
Attachment of the camera and radar to the SOLO quadcopter.3.2.

This radar is attached to a UDOO X86 single board computer. This computer is based on Quad Core 64-bit new-generation X86 processors made by Intel^®^. The attached camera and radar to the SOLO quadcopter are shown in Figure 10. A 3DR Solo Quadcopter is utilized during the flights ‘missions’. This UAV has a Pixhawk-2 autopilot with an InvenSense MPU-6000 MEMS IMU, an MS5611 barometer and a U-blox GPS. Figure 11 illustrates a block diagram of the hardware configuration.

**Figure 12:**
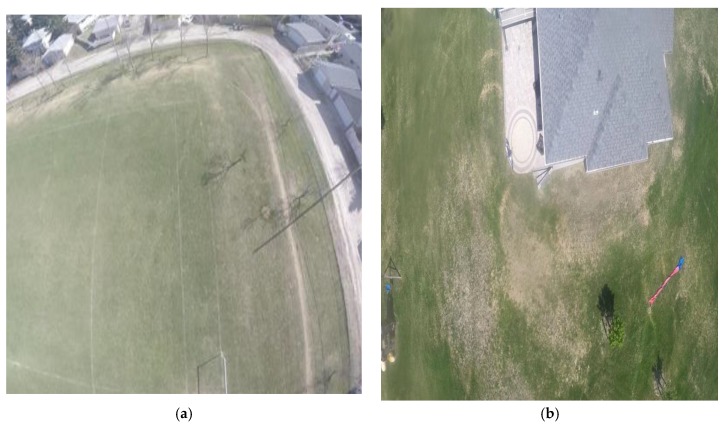
Aerial images for different flights: (**a**) first flight; (**b**) second flight.

The experiments were conducted in two different places, with different trajectories, and the radar was pitched by 60° from the quadcopter body. The first flight was performed over a football playground while the second flight was performed over multiple objects with different altitudes, such as a house, trees, grass, cars, and hangars. Figure 12 shows aerial images, which were obtained from the first and second flights.

#### 3.2.1. First Experiment

**Figure 13:**
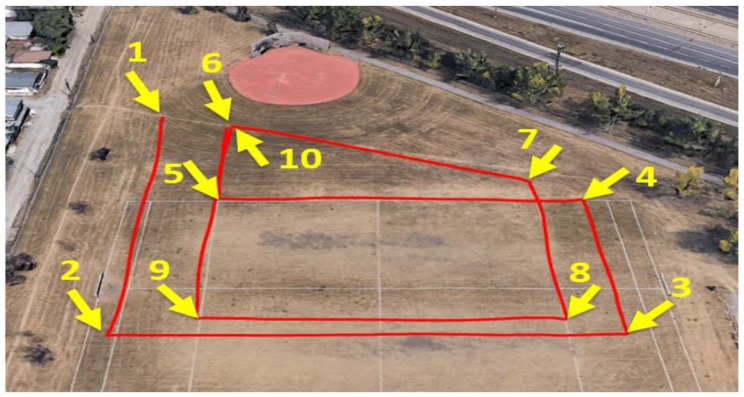
First flight trajectory.

The first flight trajectory is composed of 10 waypoints during the total flight time of 393 s with a maximum speed of 5 m/s, as shown in Figure 13.

**Figure 14:**
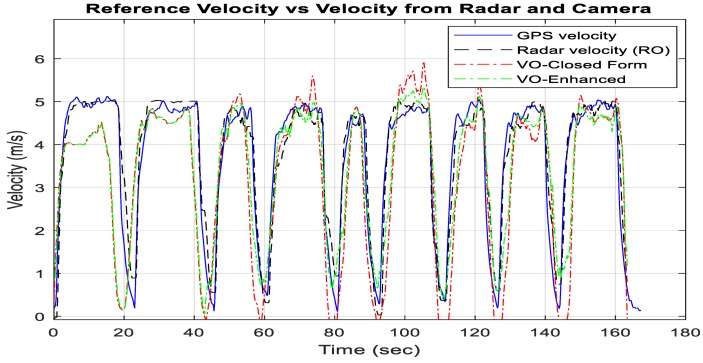
Comparisons of the forward ground truth velocity in the body frame, which are obtained from GNSS/INS integration, the estimated velocity from the RO, the typical VO, and the enhanced VO.

In Figure 14, the estimated velocity from the typical closed form monocular VO, the enhanced monocular VO, and the RO, with RMS error values of 1.29, 1.02, and 0.49 m/s, respectively, are compared to the UAV reference forward velocity in the body frame.

**Figure 15:**
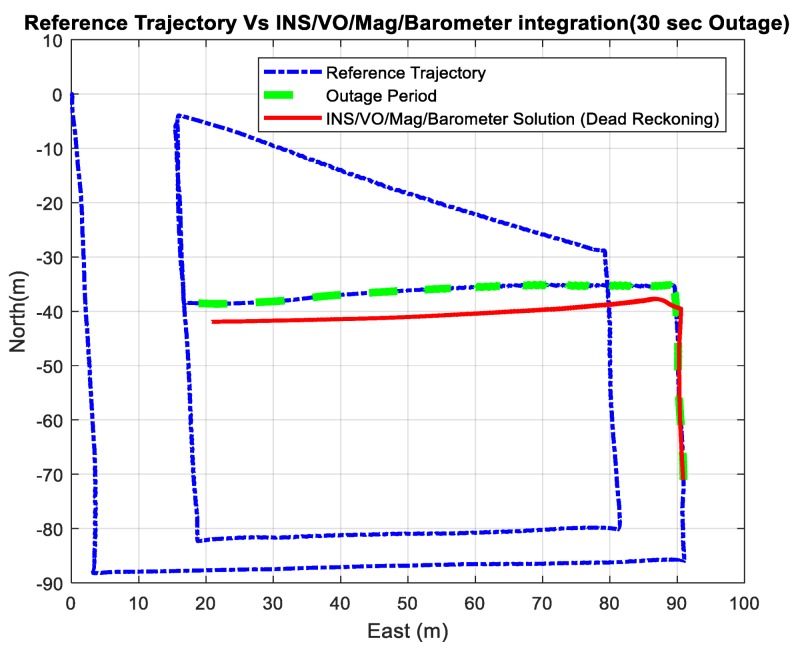
A comparison between the estimated 2D flight trajectory outage segments from the GNSS/INS integration (ground truth segment) and the enhanced monocular VO aided navigation system for 30 s.

**Figure 16:**
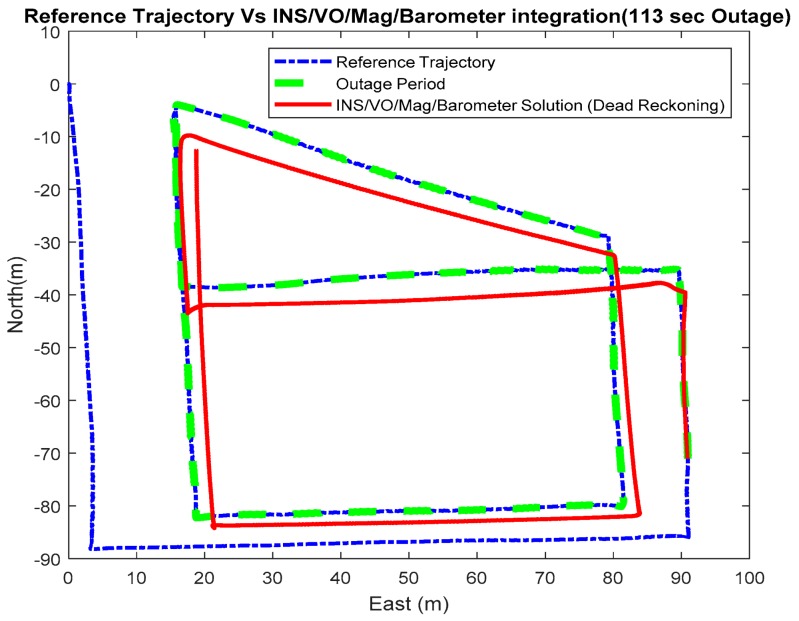
A comparison between the estimated 2D flight trajectory outage segments from the GNSS/INS integration (ground truth segment) and the enhanced monocular VO aided navigation system for 113 s.

**Figure 17:**
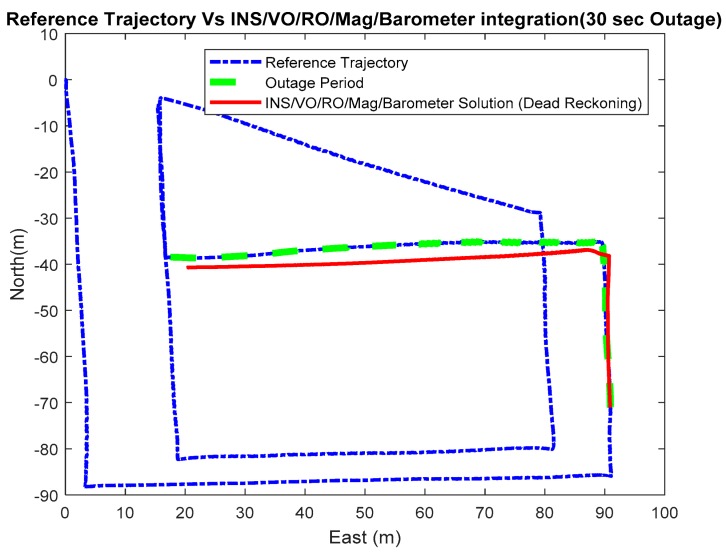
A comparison between the estimated 2D flight trajectory outage segments from the GNSS/INS integration (ground truth segment) and the integrated system aided navigation system for 30 s.

**Figure 18:**
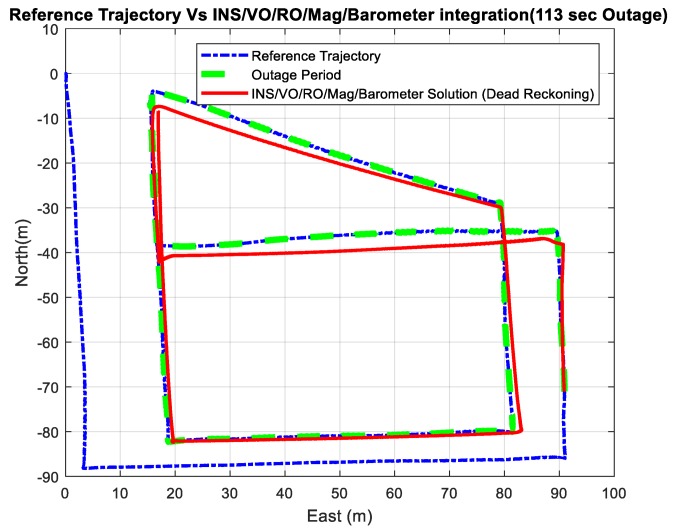
A comparison between the estimated 2D flight trajectory outage segments from the GNSS/INS integration (ground truth segment) and the integrated system aided navigation system for 113 s.

Three outage scenarios were carried, with different outage periods, ranging from 30 s to 113 s. The first outage period was performed for 30 s. Figure 15 and Figure 16 show comparisons between the estimated 2D flight trajectory outage segments from the GNSS/INS integration (ground truth segment) and an enhanced monocular VO aided navigation system during the first flight for 30 and 113 s of GNSS signal outage, respectively. Figure 17 and Figure 18 show comparisons between the estimated 2D flight trajectory outage segments from the GNSS/INS integration (ground truth segment) and the proposed integrated system aided navigation system during the first flight for 30 and 113 s of GNSS signal outage, respectively.

**Figure 19:**
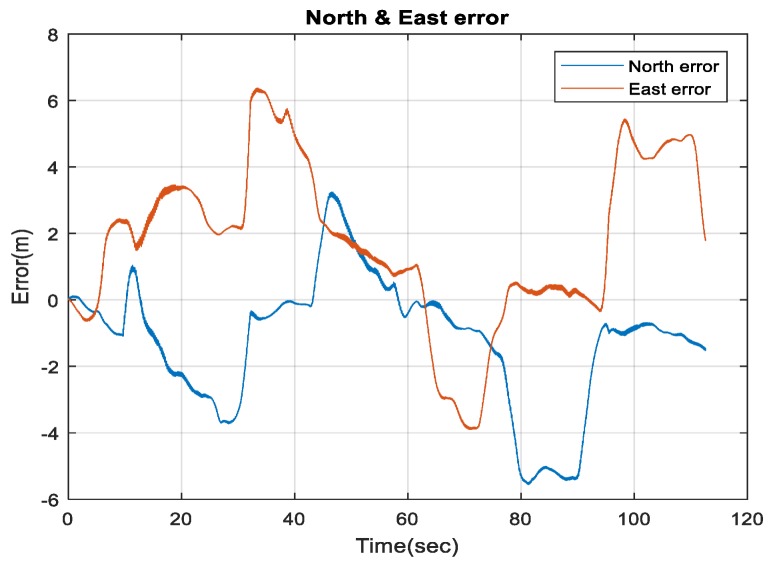
The North and East errors obtained from the proposed integrated system aided navigation system during the GNSS outage period.

Figure 19 demonstrates the ability of the proposed system (RO/enhanced monocular VO/magnetometer/barometer) to mitigate the INS drift errors when the GNSS signals get lost, and to enhance the 3D Root Mean Square Error (RMSE) positioning accuracy to be 3.2 m in 113 s.

**Figure 20:**
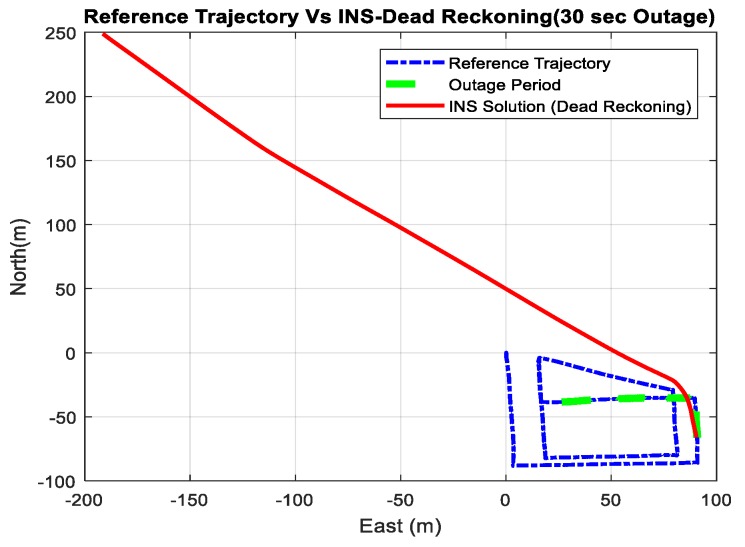
A comparison between the estimated 2D flight trajectory outage segments from the GNSS/INS integration (ground truth segment) and the integrated system aided navigation system for 30 s.

**Figure 21:**
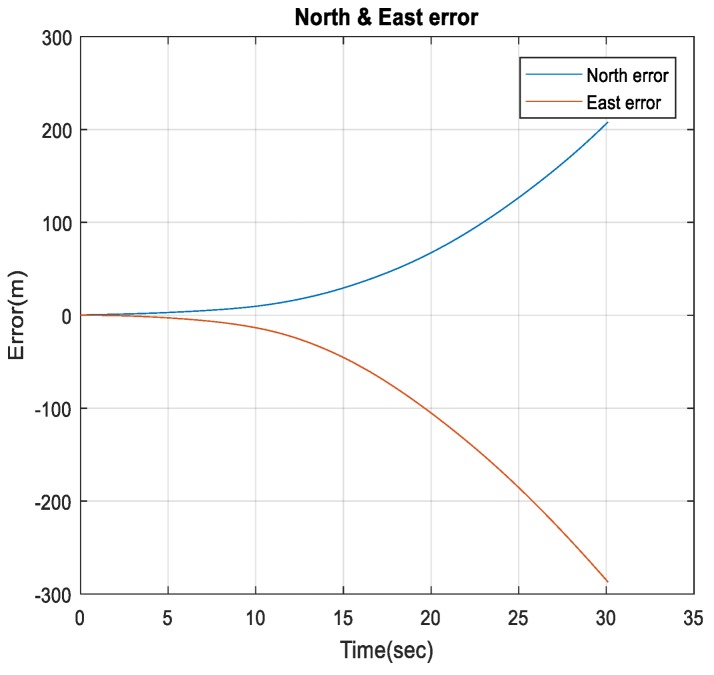
The North and East errors obtained from the INS in the standalone mode during the GNSS outage period.

Figure 20 shows a comparison between the estimated 2D flight trajectory outage segments from the GNSS/INS integration (ground truth segment) and INS in a standalone mode during the first flight for 30 s of GNSS signal outage. Figure 21 illustrates the navigation errors for the INS in the standalone mode in the North and East directions during 30 s of GNSS signal outage.

Table 1 provides comparisons of the RMS errors for the position states, which are obtained from the INS in a standalone mode, and enhanced monocular VO, and the proposed integrated system aided navigation during the GNSS outage periods. The results demonstrate the ability of the proposed integrated system to reduce the 3D positioning errors to 2.06% for 30 s, and 0.13% during 113 s of the INS drift errors in the standalone mode during the GNSS signals outages period.

**Figure 22:**
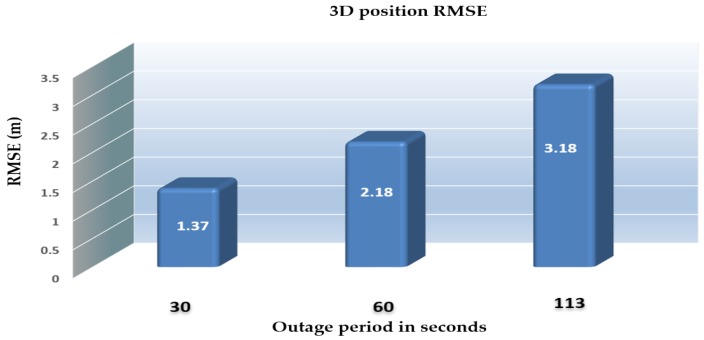
3D positioning RME errors (RMSEs) for the proposed integrated system aided navigation system during different outage periods.

The results also demonstrate the proposed integrated system’s capability of reducing the 3D positioning RMS errors to 78.04%, 71.65%, and 65.71% of the enhanced monocular VO. Figure 22 shows a comparison between the RMS errors of the 3D positioning for the proposed integrated system aided navigation during three GNSS outage periods ranged from 30 s to 113 s.

#### 3.2.2. Second Experiment

**Figure 23:**
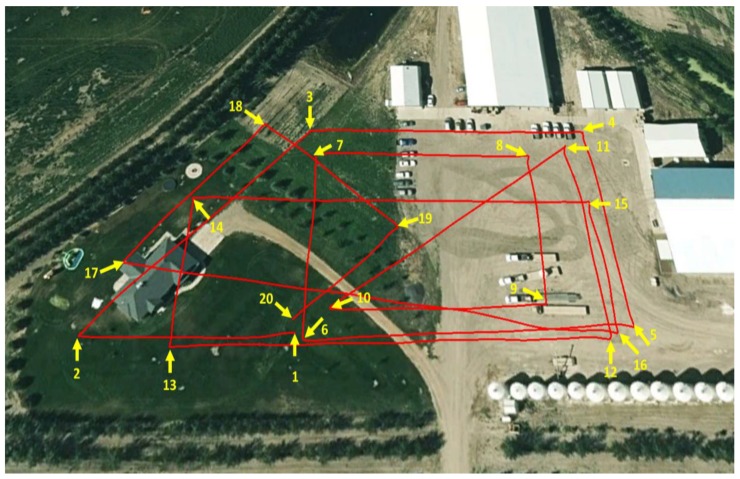
Second flight trajectory.

The second flight trajectory is composed of 18 waypoints of total flight time 393 s, with a maximum speed of 5 m/s, as shown in Figure 23.

**Figure 24:**
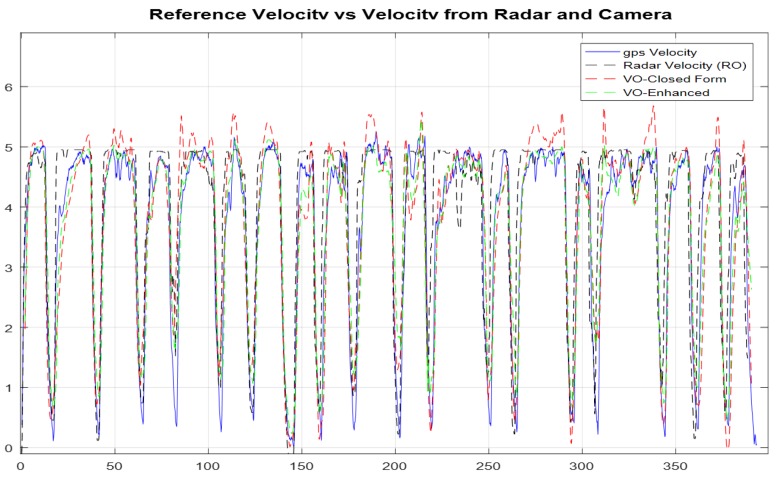
Comparisons of the forward ground truth velocity in the body frame, which are obtained from the GNSS/INS integration, the estimated velocity from the RO, the typical VO, and the enhanced VO.

In Figure 24, the estimated velocity from the typical closed form monocular VO, the enhanced monocular VO, and the RO, with RMS error values of 0.61, 0.53, and 0.75 m/s, respectively, are compared to the UAV reference forward velocity in the body frame.

The accuracy for the estimated velocity from the RO was slightly less than those of the monocular VO, and the enhanced monocular VO, because the flight was performed over multiple objects with different altitudes, ranges, and angles.

**Figure 25:**
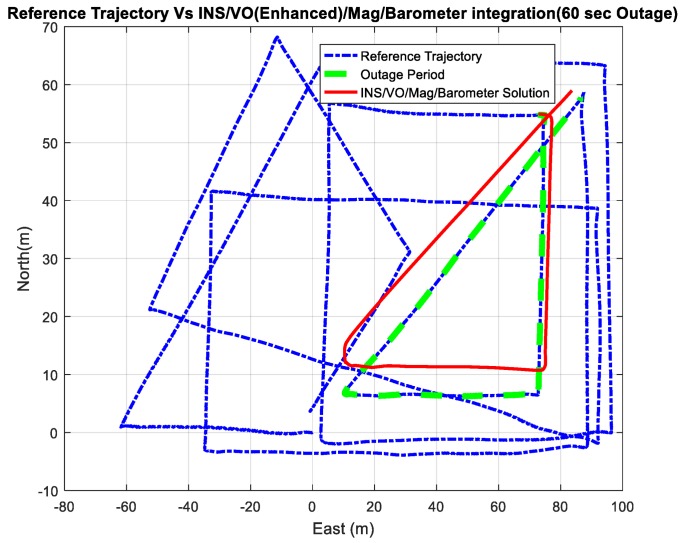
A comparison between the estimated 2D flight trajectory outage segments from the GNSS/INS integration (ground truth segment) and the enhanced monocular VO aided navigation system for 60 s.

**Figure 26:**
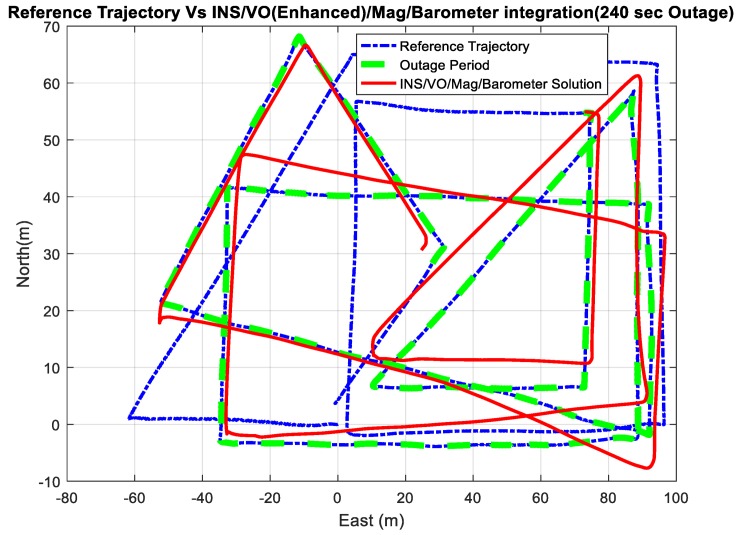
A comparison between the estimated 2D flight trajectory outage segments from the GNSS/INS integration (ground truth segment) and the enhanced monocular VO aided navigation system for 240 s.

Four outage scenarios were carried, with different outage periods, ranging from 60 s to 240 s. The first outage period was performed for 60 s. Figure 25 and Figure 26 show comparisons between the estimated 2D flight trajectory outage segments from the GNSS/INS integration (ground truth segment) and enhanced monocular VO aided navigation during the second flight for 60 s and 240 s of GNSS signal outage, respectively.

**Figure 27:**
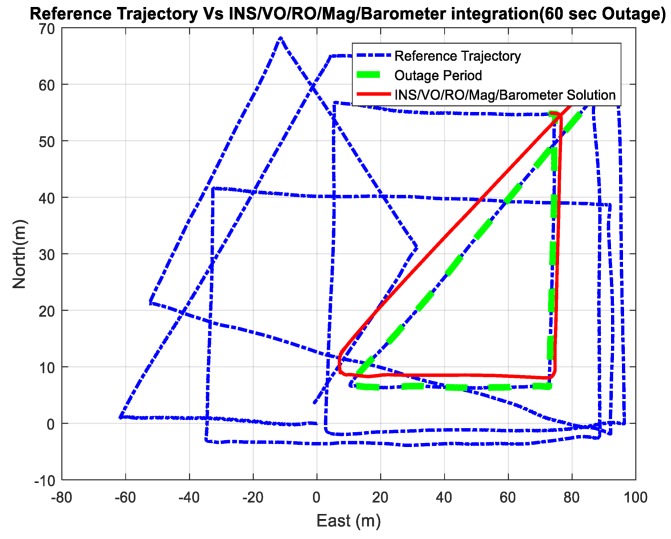
A comparison between the estimated 2D flight trajectory outage segments from the GNSS/INS integration (ground truth segment) and the integrated system aided navigation system for 60 s.

**Figure 28:**
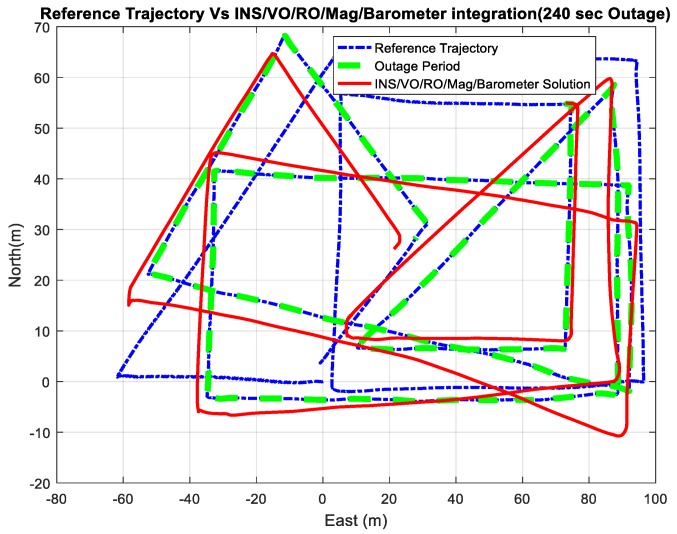
A comparison between the estimated 2D flight trajectory outage segments from the GNSS/INS integration (ground truth segment) and the integrated system aided navigation system for 240 s.

Figure 27 and Figure 28 show comparisons between the estimated 2D flight trajectory outage segments from the GNSS/INS integration (ground truth segment) and the proposed integrated system aided navigation during the second flight for 60 and 240 s of GNSS signal outage, respectively.

**Figure27.** A comparison between the estimated 2D flight trajectory outage segments from the GNSS/INS integration (ground truth segment) and the integrated system aided navigation system for 60 s.

**Figure 29:**
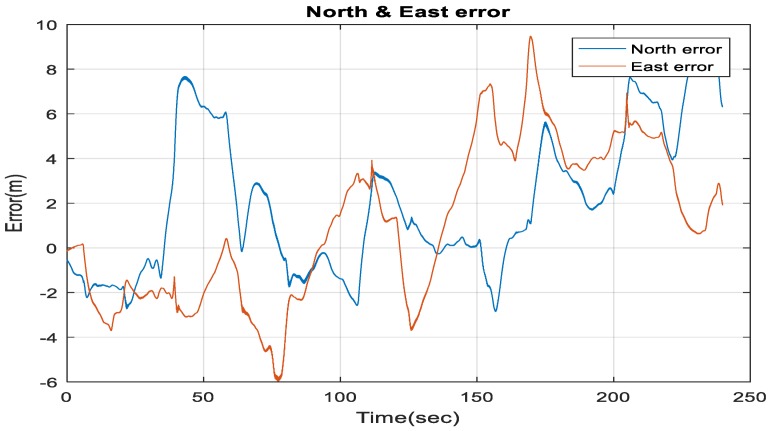
The North and East errors obtained from the proposed integrated system aided navigation system during the GNSS outage period.

Figure 29 demonstrates the ability of the proposed system (RO/enhanced monocular VO/mag/barometer) to mitigate the INS drift errors when the GNSS signals are unavailable and to enhance the 3D positioning RMSE accuracy to be 5.38 m in 240 s instead of 5878 m in the case of INS standalone solution.

**Figure 30:**
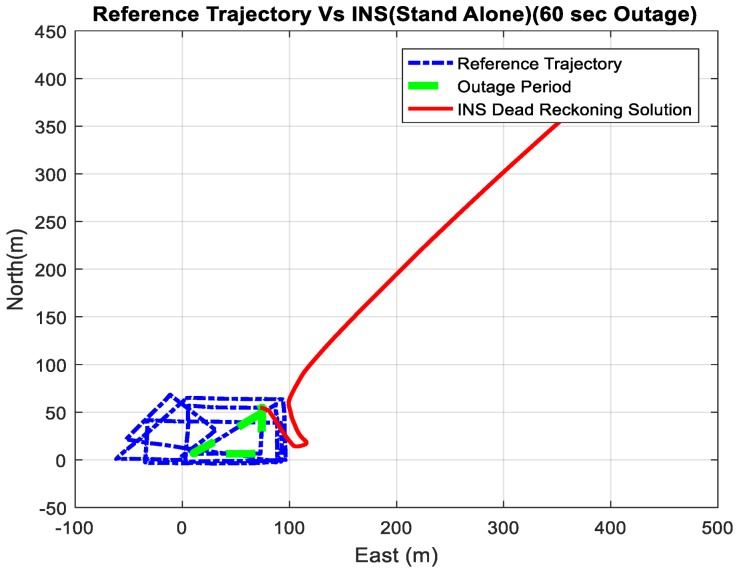
A comparison between the estimated 2D flight trajectory outage segments from the GNSS/INS integration (ground truth segment) and the integrated system aided navigation system for 60 s.

**Figure 31:**
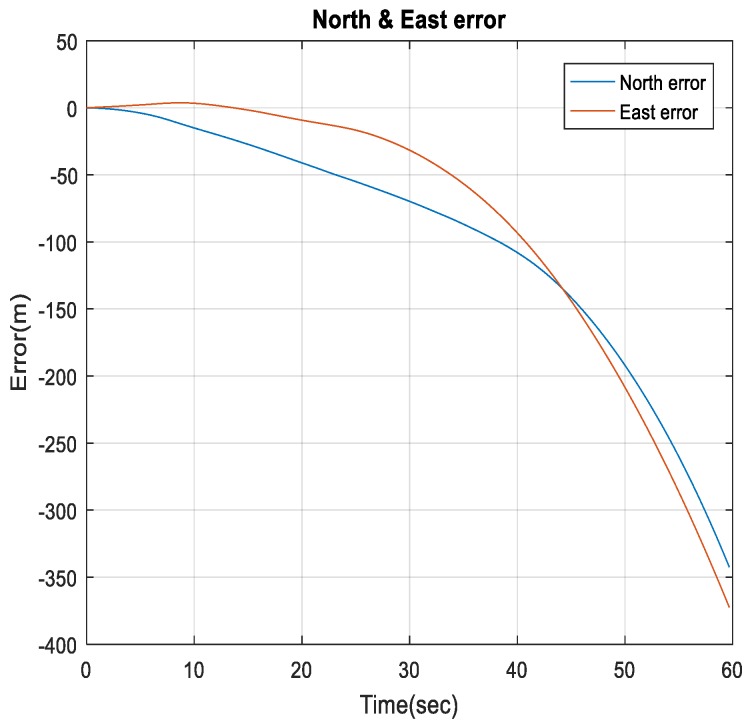
The North and East errors obtained from the INS in a standalone mode during the GNSS outage period.

Figure 30 shows a comparison between the estimated 2D flight trajectory outage segments from the GNSS/INS integration (ground truth segment) and INS in standalone mode during the second flight for 60 s of GNSS signal outage. Figure 31 illustrates the navigation errors for the INS in a standalone mode in the North and East directions during 60 s of GNSS signal outage.

Table 2 provides comparisons of the RMS errors for the position states, which are obtained from the INS in a standalone mode, and the enhanced monocular VO, and the proposed integrated system aided navigation during the GNSS outages periods. The results demonstrate the ability of the proposed integrated system to reduce the 3D positioning errors to 3.09 % for 60 s, and 0.1% during 240 s of the INS drift errors in a standalone mode during the period of GNSS signal outages.

The results demonstrate the proposed integrated system aided navigation is capable of reducing the positioning RMSEs to 92.53%, 99.41%, and 92.38% of the enhanced monocular VO during 60, 120, and 180 s of GNSS signal outages, respectively, while it has almost the same accuracy as the enhanced monocular VO during 240 s. Larger data than that for the first flight is used to train the enhanced monocular VO, which helps with an accurate estimate for the vehicle forward velocity.

**Figure 32:**
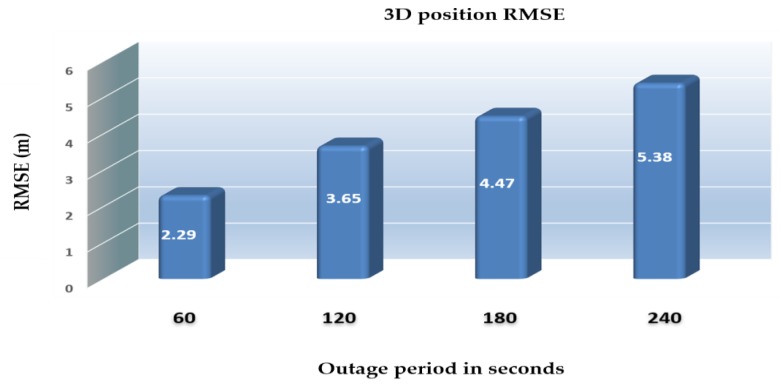
3D positioning RMSEs for the proposed integrated system aided navigation system during different outage periods.

Figure 32 shows a comparison of the 3D positioning RMSEs of the proposed integrated system aided navigation during four GNSS outage periods ranged from 60 s to 240 s.

## 4. Conclusions

An integrated navigation system for UAVs in GNSS denied environments is proposed based on micro FMCW radar and a single camera. The vehicle’s forward velocity is estimated from both the RO and the enhanced VO to enhance the INS navigation accuracy during GNSS signal outages. In addition, the estimated height from the RO is utilized to resolve the monocular VO‘s scale ambiguity. An efficient target detection approach is proposed to detect the ground objects based on a Gaussian kernel and local maxima algorithms. These detected targets are then utilized to estimate the forward velocity and the height above ground level for the vehicle. An optical flow and regression tree-based approaches are utilized to implement the enhanced monocular VO. The optical flow is employed for the forward velocity estimation purpose, while its associated drift errors are compensated based on a trained regression tree model. Theses estimated forward velocities from the RO and enhanced VO are then fused with the IMU, barometer, and magnetometer measurements via an EKF. The experimental results demonstrate the proposed system’s ability to enhance the average 3D positioning errors for the first flight by 98.65%, and to by 99.04% for the second flight with respect to the INS on the stand alone mode. In addition, it improves the average 3D positioning accuracy by 28.21% for the first flight, and 4.72% for the second flight with respect to the enhanced monocular VO.

Unlike other proposed RO methods which utilize a large unmanned aircraft or large radar or even simulate the flight missions, the proposed system utilizes a small SOLO quadcopter and a lightweight micro radar during a real flight. Such small quadcopters are typically utilized in many missions, such as search, rescue, and disaster management. The proposed algorithm has been evaluated in a generic and typical maneuvering scenario. It also avoids the various assumptions imposed by many other researches, such as straight flights, constant velocities, leveled flights, and flying over flat terrain. The RO and VO are integrated into one integrated system to help overcome their limitations. The proposed RO provides a more accurate forward velocity estimation than the enhanced monocular VO while flying over a flat terrain and it is slightly worse than the enhanced monocular VO while flying over non-flat terrain. On the other hand, the radar is immune against the environmental changes and can operate in featureless areas.

## Figures and Tables

**Table 1:** Comparison of RMS error values for the position states obtained from the INS, enhanced monocular VO aided navigation, and the integrated system aided navigation with respect to the ground truth values.

Symbol	Symbol	Outage [30 s]	Outage [113 s]
North error [m]	INS	38.06	2002
	Enhanced VO	1.52	2.64
	Integrated system	0.85	1.87
East error [m]	INS	54.59	1436
	Enhanced VO	0.85	4.02
	Integrated system	0.98	2.47
Height error [m]	INS	5.08	221
	Enhanced VO	0.25	0.61
	Integrated system	0.45	0.74
3D Position error [m]	INS	66.74	2473
	Enhanced VO	1.75	4.84
	Integrated system	1.37	3.18
Enhancement percentage from the INS%			
	Enhanced VO	97.36	99.80
	Integrated system	97.94	99.87

**Table 2:** Comparison between (RMS errors) values for the position states obtained from the INS, the enhanced monocular VO aided navigation, and the integrated system aided navigation with respect to the ground truth values.

Symbol	Symbol	Outage [60 s]	Outage [240 s]
North error [m]	INS	50.95	1233
	Enhanced VO	1.29	2.63
	Integrated system	1.57	2.75
East error [m]	INS	52.76	5680
	Enhanced VO	1.63	2.85
	Integrated system	0.85	2.81
Height error [m]	INS	12.45	878
	Enhanced VO	1.35	3.53
	Integrated system	1.44	3.68
3D Position error [m]	INS	74.39	5878
	Enhanced VO	2.47	5.24
	Integrated system	2.29	5.38
Enhancement percentage from the INS%	Enhanced VO	96.66	99.91
	Integrated system	96.91	99.90
